# Work ability and work status changes in long-term Hodgkin lymphoma survivors with focus on late adverse effects

**DOI:** 10.1007/s11764-023-01432-y

**Published:** 2023-08-01

**Authors:** Alv A. Dahl, Knut B. Smeland, Siri Eikeland, Unn-Merete Fagerli, Hanne S. Bersvendsen, Alexander Fosså, Cecilie E. Kiserud

**Affiliations:** 1 National Advisory Unit for Late Effects after Cancer Therapy, Oslo University Hospital, Oslo, Norway; 2 Department of Oncology, Oslo University Hospital, Oslo, Norway; 3 Department of Oncology, St Olav’s Hospital, Trondheim, Norway; 4 Department of Oncology, University Hospital of Northern Norway, Tromsø, Norway; 5 KG Jebsen Center for B-Cell Malignancies, University of Oslo, Oslo, Norway

**Keywords:** Work ability; Hodgkin lymphoma, Cancer survivors, Late adverse effects

## Abstract

**Purpose:**

We studied work-related issues in long-term survivors of Hodgkin lymphoma [HLSs] who had undergone treatment according to contemporary stage risk-adapted approaches. At survey, work changes and problems since diagnosis, comparisons of HLSs with low/moderate versus high work ability, associations between work issues, and late adverse effects [LAEs] were examined.

**Methods:**

This cross-sectional questionnaire-based study included HLSs treated from 1997 to 2006 and alive at the end of 2016. They completed a mailed questionnaire including work and health-related issues.

**Results:**

Among 518 invited HLSs, 297 (58%) completed the work-related issues, and 48% of them were females. Mean age at survey was 45.9 years, and mean time was 16.7 years since diagnosis. At follow-up, 71% of the HLSs held paid work and 19% were on disability pension. Only 3% of HLSs did not hold paid work at any time after diagnosis. In total, 43% HLSs had low/moderate and 57% high work ability at follow-up. Low/moderate work ability was significantly associated with older age, female sex, more LAEs, disability pension, lower household income, distressed personality, obesity, fatigue, and mental disorders. More LAEs were significantly associated with more work problems.

**Conclusions:**

Many HLSs manage to stay in the work force. Several health problems and LAEs amenable for interventions are significantly associated with low/moderate work ability and emphasize the importance of focus on these issues in long-term follow-up.

**Implications for Cancer Survivors:**

HLSs in paid work at diagnosis can be optimistic as to their future participation in work life. Screening and treatment for health problems such as LAEs may improve work ability.

## Introduction

For adults to be part of the work force is important for income, self-esteem, use of creativity and problem-solving abilities, career development, social status, and relational interactions through collaboration with colleagues. Cancer and its treatment is a well-documented cause of negative work status changes and work problems due to reduced work ability on temporary or permanent basis [1]. Work studies of cancer survivors have concerned sick leave, return to work, changes at work or of profession, reduced work ability, and rates of disability pension [1, 2]. Hodgkin lymphoma (HL) mostly affects young adults, is treated with chemotherapy and radiotherapy, and carries a good prognosis, but includes a relatively high risk for LAEs [3]i. Therefore, work-related issues are of particular interest for HL survivors (HLSs).

In studies on work issues in HLSs published between 1987 and 2017, employment rates varied between 52% and 100% [4]. In a sample of HLSs collected from 13 European countries and treated between 1964 and 2004, 70% were working at a median of 14 years after diagnosis [5]. The Scandinavian countries of Denmark, Sweden, and Norway have similar labor markets and health and welfare systems. A register-based study from Denmark [6] reported that 93% of HLSs had returned to work when assessed between five and 12 years after primary treatment. Difficulties returning to work were associated with increasing age, being female, shorter education, and drug treatment for mental health problems, but not with somatic comorbidity. In that cohort of HLSs, 9% held disability pension versus 4% in a normative sample [7], and the relative risk for such pensioning was 2.6 for the whole observation period. Factors associated with disability pensioning were the same as for impaired return to work, but also included somatic comorbidity.

A national cohort study from Sweden [8] reported an increased risk of work loss for HLSs up to 15 years after diagnosis. This loss was associated with chemotherapy, advanced stages HL, cardiovascular diseases, and secondary malignancies. Importantly, approximately 70% of the HLSs had early-stage HL in remission, and they did not experience any excess work loss except during the year of diagnosis and 1–2 years thereafter. Among studies addressing the impact of disease- and treatment-related factors including relapse, on work life issues, this study seems to be the first one to include patients treated with ABVD or BEACOPP (for explanation of abbreviations, see “Material and methods”) and limited-field radiotherapy, which are more contemporary treatment strategies, Our Norwegian research group studied post-treatment work patterns in a population-based sample of lymphoma patients treated with high-dose chemotherapy with autologous stem cell transplantation (HDT-ASCT) among whom 25% were HLSs. At a mean of 12.5 years since diagnosis, 58% were employed, and being non-employed was significantly associated with fatigue, mental distress, and type D personality [9].

In contrast to work status categories like holding paid work or being on disability pension, work ability is a dimensional concept. Work ability can be defined as the individual balance between work conditions and human resources defined by health, functional capacity, values, attitudes, and motivation [10]. Work ability was originally rated as seven dimensions by the Work Ability Index (WAI) developed by the Finnish Institute of Occupational Health [11]. Studies have shown that the total WAI score correlates highly with the dimension of “current work ability compared to highest work ability ever” rated on an 11-point Likert scale [12, 13]. Assessment of work ability is useful for at least two reasons: It can be used across professions, and it is independent of current work status. Boelhouwer et al. [14] recently published a systematic review of 36 work ability studies in cancer survivors. In general, they reported few studies assessing long-term work ability, and consistent negative associations between work ability and late adverse effects (LAEs), fatigue, and cognitive complaints among survivors. Three studies reported WAI scores in HLSs, two dimensionally [9, 15] and one as a dichotomy [16].

The current cross-sectional population-based study of HLSs is based on questionnaire data covering work-related, LAEs, health, and lifestyle characteristics together with clinical data from patients’ medical records. Besides reporting work statuses in detail, this study focuses on current work ability in HLSs exposed to treatment strategies used in recent years. Three research questions were formulated: (1) What is HLSs’ work status at follow-up, and what work changes and problems are described since diagnosis? (2) What are the differences between HLSs with low/moderate versus those with high work ability at survey? and (3) What are the associations between work-related variables and increasing number of LAEs?

## Material and methods

### Study design

This cross-sectional study collected data from a cohort of HLSs by sending them a mailed invitation and questionnaire at survey.

### Sample and treatment characteristics

This study concerned HLSs as identified by the Norwegian Cancer Registry treated from 1997 to 2006 alive and aged 8–49 years at diagnosis since they were within working age (18–68 years) at the end of 2016. Among 518 eligible HLSs, 303 responded (58% response rate), and 297 completed the work-related outcome measures of the study.

The treatment modalities were as follows [17]: Patients were treated by contemporary stage and risk-adapted strategies. From 1997, adult patients with classical HL stages I–IIA were treated with 2–4 courses of ABVD (doxorubicin, bleomycin, vinblastine, dacarbacine) followed by modified involved-field radiotherapy 30-35 Gray. Treatment of nodular lymphocyte-predominant HL in stages I–IIA consisted of 30 Gray involved-field radiotherapy, or in isolated cases of stage IA disease, surgical removal only. For stages IIB–IV, most adults received 6–8 courses of ABVD, but from 1999, high-risk patients were treated with 6–8 courses of BEACOPP (bleomycin, etoposide, doxorubicin, cyclophosphamide, vincristine, prednisone, and procarbazine). Radiotherapy could be given to sites of initial bulky mass or tumor residuals in doses of 30-40 Gray.

From 1998, children < 18 years were treated with OEPA (vincristine, etoposide, prednisone, and doxorubicin) and COPP (cyclophosphamide, vincristine, prednisone, and procarbazine) followed by involved-field radiotherapy 20-30 Gray. Salvage chemotherapy usually included ifosfamide, gemcitabine, and vinorelbine (IGEV), less often DHAP (dexamethasone, cytarabine, and cisplatin) or brentuximab vedotin, often followed by high-dose therapy with HDT-ASCT and radiotherapy.

### Work-related measures

*Work ability* was self-reported as responses to the WAI item no. 1: “Assume that your work ability at its best has a value of 10 points. How many points would you give your current work ability?” on an 11-point Likert scale from 0 (“currently not able to do work”) to 10 (“work ability as previous lifetime best”). The psychometrics of this item is well documented [12, 13]. The numeric work ability scale was rated at survey and retrospectively at diagnosis, and the difference was calculated and categorized as “worse,” “unchanged,” or “better” than at diagnosis.

The dichotomy of low/moderate versus high current work ability score as outcome variable was defined for this study as the median of the WAI item no. 1 score of the sample at survey. The scores 0–7 represented the low/moderate work ability group, and the scores 8–10 the high work ability group. To the best of our knowledge, this dichotomization of the WAI scores is unique to this study.

The *current work status* item had five response categories: employed, unemployed, on disability pension, retired, and others (students, homemakers). Holding paid work at diagnosis was coded as “yes” or “no” and being employed after diagnosis as “all the time,” “part of the time,” or “no.” Disability pension could be attained before the diagnosis of HL or between diagnosis and survey, and only the latter was deemed due to HL. *Changes of workplace after diagnosis* and eventually if due to HL were reported as “yes” or “no.” *Reduction of physical and mental work ability due to HL* was dichotomized as “present” (“to some degree,” “quite much,” and “very much”) or “absent” (“no” and “quite little”).

### Scales

All the scales of the study had established psychometric properties. Internal consistencies of scales were calculated as Cronbach’s coefficient alpha, and acceptable alpha values is between 0.66 and 0.92.To our knowledge, only the Fatigue Questionnaire has previously been used in studies specifically targeting lymphoma patients.

*Type D Personality Questionnaire (DS14).* The DS14 examines the personality traits of negative affectivity and social inhibition with seven items each [18]. Negative affectivity correlates positively with neuroticism and social inhibition negatively with the extraversion of the “Big Five” personality concept of five basic personality traits [19]. Individuals who score highly on both negative affectivity and social inhibition are considered to have distressed (type D) personality. In the general population presence of type D personality is associated with increased rates of sick leave, stress and fatigue at work [20], and higher comorbidity burden, health care utilization, mental health problems, and poorer quality of life in cancer survivors [20, 21].

Each item of the DS14 is scored from 0 (“false”) to 4 (“true”), giving sum scores on each trait from 0 to 28. Distressed personality is defined by a sum score of ≥ 10 on each trait. The Cronbach alphas were 0.89 for both negative affectivity and social inhibition in our sample.

*Fatigue Questionnaire (FQ).* The FQ consists of two subscales for physical (seven items) and mental fatigue (four items) that are added as the total fatigue score. Each item was rated from 0 (“less than before”/“not at all”) to 3 (“much more than usual”), so the total fatigue score varies from 0 to 33 with higher scores implying more fatigue [22, 23]. Alpha for total fatigue was 0.93 in our sample.

*Impact of Event Scale (IES-6).* The IES 6-item version assesses post-traumatic stress symptoms related to the HL trajectory with two items each on intrusion, avoidance, and hyperarousal. Each item is rated from 0 (“not at all”) to 4 (“very much”), providing a 0 to 24 total IES-6 severity score. A probable case of posttraumatic stress disorder (PTSD) had a sum score ≥ 9 [24]. The alpha was 0.89 in our sample.

*The Patient Health Questionnaire-9 (PHQ-9).* The PHQ-9 covered depression symptoms experienced during the last 2 weeks, and each item was scored from 0 (“not at all”) to 3 (“nearly every day”), providing a 0 to 27 severity sum score. A case of probable major depressive episode (MDE) was defined by a sum score ≥ 10 [23, 25]. Alpha was 0.87 in our sample.

*The Metamemory Questionnaire (MMQ)* covers subjective memory problems of the last week with 9 items intended to capture memory performance by their summary score. Two items cover general memory, 3 concern semantic memory, and 4 relate to working memory. Each item is scored from 1 (“no”/“never”) to 3 (“yes a lot”/“often”), and the summary score ranges from 0 to 18, with higher score implying more memory deficit [26]. Alpha was 0.88 in our sample.

### Other variables

*Sociodemographic variables.* Partnership status was either married/cohabiting or not living with a partner. Short education was defined as ≤ 12 school years completed versus long education (> 12 years). Annual household income before taxation was scored as low (≤ NOK 600,000) or high (> NOK 600,000) since the median Norwegian household income in 2016 was NOK 600,000 according to Statistics Norway Income and wealth statistics for households (https://www.ssb.no/en/inntekt-og-forbruk/inntekt-og-formue/statistikk/inntekts-og-formuesstatistikk-for-husholdninger).

*Oncological variables.* Data on histology, stage, and treatment were extracted from medical files. LAEs were self-reported based on the respondents’ personal experience. Based on the literature [27–29], 18 LAEs were listed, and we included 15 of them which were not covered by separate measures (fatigue, memory problems, and psychological reactions). Only the statement of “I have personal experience” for each LAE was considered as a positive response, and the percentages reported are given within brackets for each LAEs: hormonal changes (18%), reduced fertility (19%), cardiovascular diseases (8%), lung problems (12%), dental problems (26%), hearing problems (8%), muscular cramps (19%), nerve pains and/or numbness in hands/feet (30%), second cancer (8%), sexual problems (16%), osteoporosis (3%), lymphedema (10%), radiation injuries (26%), and other problems, to be specified (9%). The number of reported LAEs was categorized as none (reference), 1–2, and ≥ 3 LAEs. *Obesity at survey* was defined as body mass index ≥ 30 kg/m^2^.

### Statistical analyses

Missing data on scales were imputed by the mean item scores when at least half of the items were completed. Comparisons of groups of HLSs were performed with chi-squared tests for categorical variables, independent sample *t* tests for continuous variables with normal distribution, and Mann-Whitney *U* tests in case of skewed distributions. Comparisons of three groups on continuous variables were performed with one-way analysis of variance using Bonferroni’s correction. Internal consistencies of scales were calculated by Cronbach’s coefficient alpha.

Univariate and multivariable logistic regression analyses with relevant independent variables and low/moderate work ability as dependent variable with high work ability as reference were performed. Since the low/moderate work ability group consisted of 128 HLSs, we only included the 10 clinically most relevant independent variables significant in the univariate analyses, in the multivariable analysis. Depression was omitted from that analysis since the PHQ-9 total score correlated 0.75 with the total fatigue score. The strength of associations was described by odds ratios (ORs) with 95% confidence intervals (95% CI). The level of significance was set at *p* < 0.05, and all tests were two-sided. Data analyses were performed with IBM SPSS version 28.0 for PC (IBM, Armonk, NY).

## Results

### Characteristics of the total sample

As to sex, 48% of the HLSs were females. The mean age of the HLSs at diagnosis was 29.2 years (SD 9.5), at survey 45.9 years (SD 9.6), and the mean time from diagnosis to survey was 16.7 years (SD 3.0). Table 1 displays clinical data on HL, treatment, and rates of LAEs of the total sample.

**Table 1:** Descriptions of sociodemographic and oncological variables in the work ability subgroups and the total sample

Variables	Low/moderatework ability (*N* = 128)	High work ability (*N* = 169)	*p* value	Total sample (*N* = 297)
*Age at diagnosis, mean (SD)*	30.6 (9.8)	28.2 (9.1)	**0.031**	29.2 (9.5)
*Age at survey, mean (SD)*	47.8 (9.7)	44.5 (9.4)	**0.003**	45.9 (9.6)
*Follow-up time, mean (SD)*	17.3 (2.8)	16.3 (3.0)	**0.005**	16.7 (3.0)
*Sex, N (%)*			**0.011**	
Females	72 (56)	70 (41)		142 (48)
Males	56 (44)	99 (59)		155 (52)
*Histology, N (%)*			0.70	
Classical HL	117 (91)	150 (89)		267 (90)
NLPHL	10 (8)	18 (10)		28 (9)
Unclassified	1 (1)	1 (1)		2 (1)
*Stages, N (%)*			0.92	
I–IIA	78 (61)	104 (62)		182 (61)
IIB–IV	50 (39)	65 (38)		115 (39)
*B symptoms, N (%)*			0.36	
No	82 (64)	117 (69)		199 (67)
Yes	45 (35)	52 (31)		97 (33)
Unknown	1 (1)	0 (0)		1 (0)
*Treatment modalities, N (%)*				
Antracyclines	122 (95)	157 (93)	0.39	279 (94)
Chemotherapy	123 (96)	157 (93)	0.24	280 (94)
≤ 2 lines of chemotherapy	125 (98)	164 (98)	0.98	289 (98)
> 2 lines of chemotherapy	3 (2)	4 (2)		7 (2)
ABVD	131 (78)	103 (81)	0.54	234 (79)
BEACOPP	8 (6)	13 (8)	0.63	21 (7)
HDT-ACST	17 (16)	21 (15)	0.73	38 (13)
Radiotherapy	103 (81)	126 (75)	0.37	229 (77)
*Relapse, N (%)*	22 (18)	16 (10)	**0.049**	38 (13)
*Late adverse effects, N (%)*			**< 0.001**	
None	22 (17)	65 (38)		87 (29)
1–2	42 (33)	72 (43)		114 (39)
≥ 3	64 (50)	32 (19)		96 (32)

*p < 0.05

### Work characteristics of the total sample

At survey, 71% of the HLSs held paid work and 19% were on disability pension (Table 2). Since 70% reported working at diagnosis, the proportion working was similar at survey. Only 3% of HLSs did not hold paid work at any time after diagnosis, and 14% obtained their disability pension after HL treatment, which implies that 5% of the HLSs got their disability pension already before the diagnosis of HL. Better work ability at follow-up compared to diagnosis was reported by 11% of HLSs, unchanged by 33%, and worse by 56% of the HLSs. Change of workplace since diagnosis was confirmed by 57% of HLSs, and 33% of them stated that the change was due to HL. At survey, poor psychological work ability due to HL was reported by 41% of HLSs, and poor physical work ability by 50%. Among HLSs, 71% had a family income at survey above the median income of Norwegian families.

**Table 2:** Findings of the subgroups with Low/moderate and High work ability and the total sample

Variables	Low/moderate work ability (*N* = 128)	High work ability (*N* = 169)	*p* value	Total sample (*N* = 297)
*Current work status, N (%)*			**< 0.001**	
Paid work	54 (42)	156 (93)		210 (71)
Unemployed	16 (13)	2 (1)		18 (6)
Disability pension	55 (43)	2 (1)		57 (19)
Retired	3 (2)	4 (2)		7 (2)
Other	0 (0)	5 (3)		5 (2)
*Working at diagnosis, N (%)*	93 (73)	115 (68)	0.33	208 (70)
*Work ability diagnosis, mean (SD)*	8,2 (3.2)	9.2 (2.2)	**0.004**	8.7 (2.7)
*Work ability survey, mean (SD)*	3.8 (2.7)	9.3 (0.9)	**< 0.001**	6.9 (3.3)
*Work ability changes, N (%)*			**< 0.001**	
Better	8 (6)	24 (15)		32 (11)
Unchanged	12 (10)	82 (51)		94 (33)
Worse	103 (84)	56 (34)		159 (56)
*Paid work after diagnosis, N (%)*			**< 0.001**	
All the time	60 (48)	133 (81)		193 (67)
Part of the time	55 (44)	31 (19)		86 (30)
No	10 (8)	0 (0)		10 (3)
*Disability pension post-treatment, N (%)*	37 (29)	5 (3)	**< 0.001**	42 (14)
*Changed work place, N (%)*	73 (57)	95 (56)	0.89	168 (57)
*Changed due to cancer, N (%)*	36 (51)	18 (19)	**< 0.001**	54 (33)
*Poor psychic work ability due to HL, N (%)*	85 (70)	32 (19)	**< 0.001**	117 (41)
*Poor physical work ability due to HL, N (%)*	109 (89)	38 (22)	**< 0.001**	147 (50)
*Household income, N (%)*			**< 0.001**	
≥ NOK 600,000	68 (54)	140 (83)		208 (71)
< NOK 600,000	57 (46)	28 (17)		85 (29)
*Type D personality, N (%)*	59 (46)	23 (14)	**< 0.001**	82 (28)
*Probable cases of MDE, N (%)*	51 (41)	17 (10)	**< 0.001**	68 (23)
*Probable cases of PTSD, N (%)*	56 (47)	27 (17)	**< 0.001**	83 (30)
*Total fatigue, mean (SD)*	19.5 (5.8)	12.7 (4.1)	**< 0.001**	15.6 (5.9)
*Partnered relationship, N (%)*	97 (76)	142 (84)	0.08	239 (81)
*Level of education, N (%)*			**< 0.001**	
Short (< 12 years)	74 (58)	57 (34)		131 (44)
Long (≥ 12 years)	54 (42)	112 (66)		166 (56)
*Obesity (BMI ≥ 30), N (%)*	39 (31)	26 (15)	**0.002**	65 (22)
*Metamemory score, mean (SD)*	17.3 (4.1)	14.2 (3.7)	**< 0.001**	15.6 (4.1)
Females	17.8 (4.2)	14.6 (3.6)	**< 0.001**	16.3 (4.2)
Males	16.7 (3.9)	13.9 (3.7)	**< 0.001**	14.9 (4.0)

*p < 0.05

### Comparisons of the low/moderate and the high work ability groups

Based on our definitions, 128 HLSs belonged to the low/moderate and 169 to the high work ability groups. At survey, the low/moderate work ability group had significantly higher mean age and mean time from diagnosis to survey. That group also had significantly higher proportions of females, HL relapse, and ≥ 3 LAEs, while other oncological variables like B-symptoms and treatment modalities showed no significant between-group differences (Table 1).

The low/moderate group also had significantly higher proportions of HLSs on disability pension, poorer physical and psychological work ability due to HL, distressed (type D) personality, obesity, probable cases of MDE, PTSD, and higher mean level of fatigue and memory problems. That group also had significantly lower proportions of HLSs with long education, holding paid work at survey, high household income, and their reported mean work ability at diagnosis was lower (Table 2).

### Univariate and multivariable analyses

The univariate analyses with low/moderate work ability as dependent variable confirmed the significant between-group differences reported in Tables 1 and 2 (Table 3). Older age at follow-up, ≥ 3 LAEs, low household income, presence of type D personality, increased level of total fatigue, and obesity remained significantly associated low/moderate work ability at survey in the multivariable analysis.

**Table 3:** Univariate and multivariable logistic regression analyses of independent variables and low/moderate work ability [high as reference] at survey as dependent variable

Variables	Univariate analyses			Multivariable analysis		
	OR	95% CI	*p* value	OR	95% CI	*p* value
Age at survey	1.04	1.01–1.06	**0.003**	1.05	1.01–1.09	**0.018**
Follow-up time	1.12	1.03–1.21	**0.006**	-	-	**-**
Relapse	1.99	0.99–3.96	0.05	-	-	-
Late adverse effects			**< 0.001**			**0.011**
None [reference]	1.00	-		1.00	-	-
1–2	1.72	0.93–3.19	0.08	0.85	0.36–2.01	0.71
≥ 3	5.91	3.10–11.24	**< 0.001**	2.94	1.17–7.43	**0.022**
Female [male reference]	1.82	1.14–2.89	**0.012**	1.72	0.84–3.51	0.14
Short education	2.69	1.68–4.33	**< 0.001**	1.56	0.75–3.23	0.24
Metamemory score	1.23	115–1.31	**< 0.001**	0.99	0.90–1.10	0.99
Work ability at diagnosis	0.87	0.80–0.96	**0.004**	-	-	-
Low household income	4.19	2.45–7.17	**< 0.001**	3.08	1.44–6.61	**0.004**
Cases of depression	6.00	3.24–11.10	**< 0.001**	-	-	-
Cases of PTSD	4.25	2.45–7.34	**< 0.001**	0.85	0.35–2.07	0.85
Type D personality	2.65	1.95–2.59	**< 0.001**	3.20	1.38–7.46	**0.007**
Total fatigue score	1.31	1.23–1.39	**< 0.001**	1.25	1.16–1.36	**< 0.001**
Obesity	2.44	1.39–4.28	**0.002**	2.72	1.19–6.23	**0.018**

*p < 0.05

### Relationships between LAEs and work variables

Table 4 displays the relationships between the three levels of LAEs and work-related variables at follow-up. The group of HLS with ≥ 3 LAEs showed a significantly lower proportion in paid work and a higher proportion on disability pension compared to the two groups with less than three LAEs. The group with ≥ 3 LAEs also had significantly lower mean work ability and comprised more HLSs on disability pension after HL treatment. In addition, HLSs in this group reported a significantly higher proportion with worse work ability at survey compared to diagnosis, and poorer physical and psychic work ability due to HL.

**Table 4:** The relationship between the three levels of late adverse effects (LAEs) and work-related variables

Variables	No LAEs (*N* = 87)	1–2 LAEs (*N* = 114)	≥ 3 LAEs (*N* = 96)	*p* values
*Current work status, N (%)*				**0.002**
Paid work	70 (82)	85 (75)	55 (58)	**0.001**
Unemployed	3 (3)	8 (6)	7 (7)	0.48
Disability pension	8 (9)	17 (15)	32 (33)	**< 0.001**
Retired	3 (3)	2 (2)	2 (2)	0.72
Other	3 (3)	2 (2)	0 (0)	0.50
*Working at diagnosis, N (%)*	61 (73)	77 (68)	70 (74)	0.63
*Work ability at diagnosis, mean (SD)*	9.3 (1.8)	8.4 (2.9)	8.5 (3.1)	**0.001**
*Work ability at survey, mean (SD)*	8.3 (2.7)	7.4 (3.0)	5.2 (3.4)	**< 0.001**
*Paid work after diagnosis, N (%)*				0.12
All the time	62 (74)	78 (70)	53 (57)	
Part of the time	21 (25)	30 (27)	35 (38)	
No	1 (1)	4 (3)	5 (5)	
*Disability pension after treatment, N (%)*	9 (10)	9 (8)	24 (25)	**< 0.001**
*Changed work place, N (%)*	45 (52)	70 (61)	53 (55)	0.37
*Changed due to HL, N (%)*	9 (10)	25 (22)	20 (21)	0.08
*Work ability changes, N (%)*				**< 0.001**
Better	6 (7)	20 (18)	6 (7)	**0.014**
Unchanged	47 (56)	32 (29)	15 (16)	**< 0.001**
Worse	31 (37)	57 (52)	71 (77**)**	**< 0.001**
Low household income, *N* (%)	18 (21)	38 (33)	29 (31)	0.14
Poor psychic work ability due to HL, *N* (%)	18 (21)	54 (49)	71 (76)	**< 0.001**
Poor physical work ability due to HL, *N* (%)	22 (25)	46 (45)	79 (76)	**< 0.001**

*p < 0.05

## Discussion

At follow-up, 71% of the HLSs held paid work and 19% were on disability pension. The proportion of HLSs working was unchanged from diagnosis to survey. Better work ability was reported by 11% of HLSs, unchanged by 33%, and worse by 56% compared to the work ability recalled from the time of diagnosis. Most HL-related variables did not differ between the low/moderate and high work ability groups, but the former group showed significantly more LAEs. In multivariable analysis, older age at follow-up, ≥ 3 LAEs, presence of type D personality, increased level of fatigue, low household income, and obesity remained significantly associated with low/moderate work ability. We observed significant associations between increasing number of LAEs and several worse work-related variables.

We find it of interest that the proportion of HLSs working was unchanged from diagnosis to follow-up although the sample had become close to 17 years older. This is particularly noteworthy since 56% of HLSs reported lower work ability at follow-up compared to what was recalled from the time of diagnosis. An interpretation could be that many HLSs worked harder with less work ability at survey to retain a paid work position. This interpretation is supported by other studies of work issues in cancer survivors [30]. Another plausible explanation could be that the findings are due to recall bias since the participants may have over-estimated their work ability at a mean of 17 years before the survey.

A Danish study reported that 93% of patients with HL returned to work after treatment [6]. This result is comparable with our finding that 97% of HLSs worked part time or full time at least for some periods after diagnosis. Becoming a HLSs influenced the rate of job changes, and the HL reported reduced physical and psychological work ability in 40 to 50% of our sample. We do not have normative data that could bring these figures into a broader work life perspective.

Of interest, 71% of HLSs had a household income at survey above the median income of Norwegian households, but since 81% of HLSs had partners, we cannot tease out the contributions made by HLSs. There are several papers on income reductions [31, 32] and financial difficulties [33] in cancer survivors. Based on our findings, a message to be conveyed to HL patients at diagnosis could be that many will be able to hold paid work and a good household income for years after treatment.

Our current work ability measure is a subjective concept rated by the patients based on their “lifetime best” level of work ability. As indicated, this evaluation could be subject to recall bias, but on the other hand, it seems plausible to remember the time when an individual was most successful in their career. To our knowledge, only one study has previously dichotomized the work ability score into two categories. Without giving any reason, Torp et al. [16] chose the same cutoff (0–7 versus 8–10) as we did based on the median score in our sample. Our justification was that we found it clinically meaningful to compare half our sample with lower score to the half with higher.

An advantage of work ability is its independence of current work status, and thereby even HLSs on disability pension or in retirement could score their work ability. Individuals outside work life also do work in their daily life, hobbies, charity, and family and social life. In our view, such considerations render the work ability concept clinically relevant for all cancer survivors. In relation to the recent review of work ability in cancer survivors [14], findings of LAEs, fatigue, memory problems, and negative associations with work ability were confirmed in our study, and the burden of LAEs and level of fatigue among HLSs were significant in our multivariable analysis.

Few studies included in the review of work ability in cancer survivors [14], however, had a follow-up time of > 2 years since diagnosis, while in our sample, mean follow-up time was 16.7 years. In this aspect, our long-time findings of work ability in HLSs represent new knowledge. We note that except for relapse and number of LAEs, the oncological characteristics and treatment modalities of HL showed no significant associations with current work ability.

Fatigue and depression are well-known problem of survivors of many types of cancer [29]. In our sample, we found levels of total fatigue and probable cases of MDE that were significantly higher than in the general Norwegian population [23]. In univariate analyses, both these variables were significantly associated with low/moderate work ability, and total fatigue score even in the multivariate analysis, where depression was excluded due to multicollinearity. The association with HL-related traumatic anxiety was also significant in univariate analysis. The rate of probable PTSD was 30% by self-rating in our HLS sample and 3% in a normative Norwegian sample diagnosed by interviews [34]. Even with reservation for variation in methods, the difference appears highly significant.

Cognitive complaints is a common LAE in survivors who had chemotherapy [29], and in our HLS sample, the women showed significantly higher mean score on subjective memory impairment compared to normative data (27) [*N* = 141, mean 16.2 (SD 4.2) versus *N* = 20,792, mean 13.9 (SD 3.0), *p* < 0.001], while no significant difference was observed for men [*N* = 152, mean 14.9 (SD 4.0) versus *N* = 16,613, mean 14.5 (SD 3.2), *p* = 0.12]. Recently, personality has been identified as a background factor for mental distress, comorbidity, and LAEs in cancer survivors [35–37]. The relevance of type D personality for work problems in general [38] was expanded by our findings in HLSs.

Fatigue can be understood as both physical and mental, while depression, anxiety, memory problems, and personality are primarily mental, and these findings confirm the relevance of mental health issues for work ability in long-term HLSs.

We observed a significant association between the number of LAEs and the level of work ability at follow-up. This finding underlines the importance of paying attention to LAEs for those providing healthcare to HLSs and possibly motivates follow-up care to enhance the education of patients on LAEs, as previously suggested [36].

The policy of the Norwegian government is that individuals who have reasonable work ability should stay in their jobs if possible. Also, healthcare providers following HLSs should try to preserve and improve work ability from diagnosis and through treatment and the years to follow.

Apart from LEA and fatigue, we found that low/moderate current work ability was associated with several other variables that could be amenable to intervention by healthcare providers, and eventually could improve current work ability. Such interventions could be based on identification of traumatic anxiety, memory impairment, depression, type D personality, and obesity with information from simple questions or screening questionnaires. Obesity is amenable to lifestyle interventions, while traumatic anxiety and depression can be treated with medication and/or psychotherapy. Memory training in cancer survivors with cognitive problems is a field in rapid development [37]. Dysfunctional personality traits have recently been shown to be more amenable to interventions than previously believed [39], suggesting increased clinical utility for screening tests for personality traits during the cancer trajectory. The DS14 is an example of such a screening instrument [18]. Healthcare providers should eventually seek support from specialists, as this can facilitate work reintegration.

Strengths of our study are the considerable sample size of HLSs at long-term after diagnosis and use of established instruments with good psychometric properties. One limitation of our study is the cross-sectional design, and that we thereby lack pre-treatment data on work ability, relying on retrospectively collected information. Such information can be weakened by recall bias. Prospective studies of work ability in HLSs are needed to understand the development of work ability after diagnosis and treatment. Reference data on current work ability in the general population is also needed. As previously reported, non-responders in this study were generally younger and more often male, but in lack of other data from the non-responders, we were not able to do any further attrition analysis for the outcomes of this report, comparing the responders to total sample invited to participate.

## Conclusions

Although work ability frequently was reduced since diagnosis, 71% of HLSs held paid work close to 17 years after diagnosis. Work ability is a useful measure for the working capacity of HLSs independent of their work status. Multivariable analysis showed that several modifiable factors were related to low/moderate work ability at long-term survey. Particularly, an increased rate of LAEs is significantly associated with poorer work outcomes. These factors should be evaluated repeatedly during the trajectory of HLSs.

## Data Availability

According to Norwegian data legislation, the data of this study cannot be made available. Requests may be addressed to the senior author.

## Abbreviations

95% CI: 95% Confidence intervals
ABVD: Combination of doxorubicin, dakarbazin, vinblastine, and bleomycin
BEACOPP: Combination of doxorubicin, cyclophosphamide, etoposide, vincristine, bleomycin, prokarbazin, and prednisolone
CI: Confidence interval
DS14: Distressed personality test
FQ: Fatigue Questionnaire
HDT-ACST: High-dose chemotherapy and autologous stem-cell transplantation
HL: Hodgkin’s lymphoma
HLSs: Hodgkin’s lymphoma survivors
IES-6: Impact of Event Scale, 6-item version
LAEs: Late adverse effects
MDE: Major depressive episode
MMQ: Metamemory Questionnaire
OR: Odds ratio
PHQ-9: Patient Health Questionnaire, 9-item version
PROM: Patient-rated outcome measure
PTSD: Posttraumatic stress disorder
SD: Standard deviation
WAI: Work Ability Index
